# Increases in Doublecortin Immunoreactivity in the Dentate Gyrus following Extinction of Heroin-Seeking Behavior

**DOI:** 10.1155/2012/283829

**Published:** 2012-11-01

**Authors:** Megan P. Hicks, Kelly C. Wischerath, Amber L. Lacrosse, M. Foster Olive

**Affiliations:** ^1^Department of Psychiatry and Behavioral Sciences, Medical University of South Carolina, Charleston, SC 29425, USA; ^2^Department of Psychology, Arizona State University, 950 S. McAllister Avenue, P.O. Box 871104, Tempe, AZ 85287, USA; ^3^Interdisciplinary Graduate Program in Neuroscience, Arizona State University, Tempe, AZ 85287, USA

## Abstract

Adult-generated neurons in the dentate gyrus (DG) of the hippocampus play a role in various forms of learning and memory. However, adult born neurons in the DG, while still at an immature stage, exhibit unique electrophysiological properties and are also functionally implicated in learning and memory processes. We investigated the effects of extinction of drug-seeking behavior on the formation of immature neurons in the DG as assessed by quantification of doublecortin (DCX) immunoreactivity. Rats were allowed to self-administer heroin (0.03 mg/kg/infusion) for 12 days and then subjected either to 10 days of extinction training or forced abstinence. We also examined extinction responding patterns following heroin self-administration in glial fibrillary acidic protein thymidine kinase (GFAP-tk) transgenic mice, which have been previously demonstrated to show reduced formation of immature and mature neurons in the DG following treatment with ganciclovir (GCV). We found that extinction training increased DCX immunoreactivity in the dorsal DG as compared with animals undergoing forced abstinence, and that GCV-treated GFAP-tk mice displayed impaired extinction learning as compared to saline-treated mice. Our results suggest that extinction of drug-seeking behavior increases the formation of immature neurons in the DG and that these neurons may play a functional role in extinction learning.

## 1. Introduction

Adult-born neurons in the dentate gyrus (DG) of the adult hippocampus are generated from neural progenitor cells (NPCs) located in the subgranular layer of this brain region, which proliferate and differentiate first into immature neurons and subsequently migrate into the granule cell layer as mature neurons where they integrate into existing hippocampal circuits [1]. There are numerous lines of evidence to support the notion that mature adult-born neurons contribute to several forms of hippocampus-dependent learning and memory processes such as spatial navigation, contextual learning, and pattern separation (reviewed in [2–7]). Various neuropsychiatric disorders such as depression, schizophrenia, and drug addiction are characterized by alterations in neurogenesis in the DG [1, 8–10]. In the drug addiction literature, there is general agreement that most drugs of abuse, including opiates, ethanol, and psychostimulants, suppress neurogenesis in the DG [11–16], and it has been suggested that diminished DG neurogenesis may play a causative role in certain cognitive deficits frequently observed in drug addicts, such as maladaptive contextual and episodic memory formation, altered spatial abilities, cognitive inflexibility, and susceptibility to relapse [17–19].

 While the majority of research on neurogenesis in the adult DG has focused on factors influencing the proliferation, differentiation, and survival of NPCs, as well as the role of mature adult-born neurons in various cognitive functions, relatively little is known about the function of adult-born neurons that have not yet fully developed into mature granule cells. There are known species-specific time periods (approximately 3–6 weeks in rodents) between NPC proliferation and formation of mature dentate granule cells when adult-born neurons are considered immature. During this time period, immature adult-born neurons undergo a substantial amount of morphological and physiological maturation and exhibit enhanced synaptic plasticity and lower thresholds for the induction of long-term potentiation (LTP) [20, 21]. During this stage, immature neurons transiently express the microtubule-associated protein doublecortin (DCX) [22, 23], which allows for immunohistochemical detection and quantification of the number of immature neurons in the DG. Recent studies using novel transgenic approaches to selectively ablate immature neurons in the DG have revealed that in fact these neurons mediate spatial memory as well as the extinction of conditioned fear [24]. However, the role of immature DG neurons in extinction of addiction-related behaviors has not yet been explored.

 Given that extinction is an established form of new and active learning [25–28], the goal of the present study was designed to explore the possibility that immature DG neurons are involved in the extinction of drug-seeking behavior. In Experiment 1, we sought to determine if extinction of drug-seeking behavior following heroin self-administration would alter DCX immunoreactivity in the DG as compared to animals undergoing forced abstinence. In Experiment 2, we sought to determine if suppression of the generation of immature neurons in the DG would affect extinction responding following heroin self-administration.

## 2. Material and Methods

### 2.1. Subjects

Animals were maintained on a 12-hour light-dark cycle (lights off at 07:00 hr) in a temperature- and humidity-controlled room. Animals were given ad libitum access to food and water during all phases of the experiment except during behavioral testing. All experimental procedures were conducted with the approval of the Institutional Animal Care and Use Committee of the Medical University of South Carolina and in accordance with the Principles of Laboratory Animal Care and the Guidelines for the Care and Use of Mammals in Neuroscience and Behavioral Research (National Research Council, 2003). For Experiment 1, male Sprague-Dawley rats (Harlan Laboratories, Livermore, CA, USA) weighing approximately 250–275 g were individually housed upon arrival. For Experiment 2, male GFAP-tk mice (B6.Cg-Tg(Gfap-Tk) 7.1 Mvs/J, stock #005698; Jackson Laboratories, Bar Harbor, ME, USA) weighing approximately 20–25 g were individually housed upon arrival. This mouse strain was generated as described elsewhere [29, 30] to express a herpes simplex virus thymidine kinase (tk) exclusively in GFAP-positive cells, and proliferating cells expressing the transgene in the presence of the antiviral agent ganciclovir (GCV) produce toxic nucleotide analogues that promote cell death. Thus, GFAP-tk mice have been used as a tool for investigating the function of adult-generated neuronal precursors [31]. This strain has been backcrossed from the founder line onto a C57BL/6 background strain for more than 12 generations. Prior to shipment, all animals were implanted with jugular vein catheters by Harlan or Jackson Laboratories Surgical Services. Catheters were filled with a Hep-Lock solution, tunneled subcutaneously to exit the dorsum between the scapulae, plugged with 1 cm segments of stainless steel tubing and secured to skin on the dorsum with wound clips. 

### 2.2. Drugs

Heroin (diacetylmorphine hydrochloride), 5-bromodeoxyuridine (BrDU), and sodium pentobarbital were obtained from Sigma-Aldrich (St. Louis, MO, USA). Heroin was dissolved in sterile saline for intravenous (i.v.) self-administration. BrDU was dissolved in sterile saline at a concentration of 50 mg/mL for intraperitoneal (i.p.) administration and was administered in a volume of 1 mL/kg to yield a final dose of 50 mg/kg per injection. Sodium pentobarbital was dissolved in sterile saline containing 10% v/v ethanol and 40% v/v propylene glycol at a concentration of 150 mg/mL for i.p. administration. Ganciclovir (GCV) was obtained from Waterstone Technology (St. Carmel, IN, USA) and dissolved at a concentration of 10 mg/mL in sterile saline for subcutaneous administration via osmotic minipumps. 

### 2.3. Apparatus

Drug self-administration and extinction training were conducted in operant self-administration chambers (ENV-008 for rats, ENV-307 for mice; Med Associates, St. Albans, VT, USA). Each self-administration chamber was located inside a sound-attenuating cubicle equipped with a house light and an exhaust fan designed to mask external noise and odors and was interfaced to a PC computer. Positioned above each lever was a 2.5 cm diameter white stimulus light. Syringe pumps were located outside self-administration chambers and were interfaced to a PC. Drug solutions were delivered via the syringe pump through a single-channel liquid swivel mounted atop of the chamber, which was connected to a vascular access port.

### 2.4. Surgical Procedures

Upon arrival, animals were allowed one day of acclimation before surgical procedures. For Experiment 1, rats were anesthetized with isoflurane (2% v/v) vaporized in medical-grade oxygen at a flow rate of 2 L/min. Staples surrounding catheter tubing were removed and a one-inch longitudinal incision was made for implantation of a threaded vascular access port (Plastics One; Roanoke, VA, USA). Access ports were attached to a mesh collar that was sutured underneath the surrounding tissue within the incision and were sealed with a piece of Tygon tubing closed at one end and a protective cap. Following surgical procedures, rats were given 5 days of postoperative care during which they received daily intravenous infusions of 70 U/mL heparin (0.2 mL volume) to maintain catheter patency and 100 mg/mL cefazolin (0.1 mL volume) to protect against infection. Rats also received daily subcutaneous injections of 2.5 mg/mL of meloxicam (0.15 mL volume) for surgery-related discomfort. The surgery site was also treated with topical lidocaine and triple antibiotic ointment to facilitate healing of the wound.

 For Experiment 2, mice were anesthetized as described above, the catheter exit wound was enlarged to 1 cm in length, and mice were implanted with sterile osmotic minipumps (Model 1004, pump rate 0.11 *μ*L/hr; Alzet; Cupertino, CA, USA) containing either sterile saline or GCV (10 mg/mL) into the dorsum. The catheter was trimmed to extend 1 cm from the exit wound. Following minipump implantation, mice were treated with 10 U/mL heparin (0.05 mL volume) to maintain catheter patency and 10 mg/mL cefazolin (0.05 mL volume) to protect against infection. The surgery site was treated with topical lidocaine and triple antibiotic ointment to facilitate healing of the wound, and mice were allowed 1 day to recover prior to initiation of drug self-administration procedures.

### 2.5. Heroin Self-Administration

**Figure 1:**
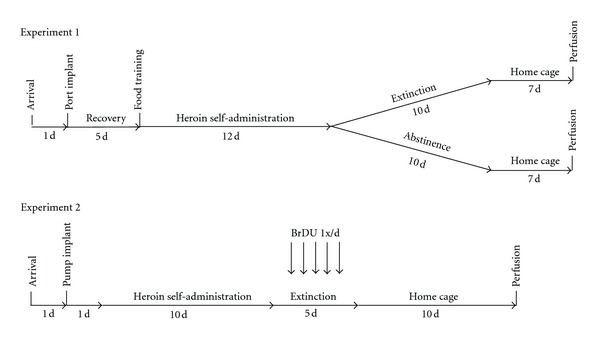
Timelines of procedures in Experiments 1 and 2.

All self-administration procedures were conducted during the dark phase of the light-dark cycle. A timeline of both experiments is provided in Figure 1. In Experiment 1, to initiate operant responding, rats were placed in the self-administration chambers for a single 16 hr overnight training sessions whereby each press on the designated active lever delivered a 45 mg food pellet (Test-diet, Richmond, IN, USA) into a pellet receptacle on a fixed-ratio 1 (FR1) schedule of reinforcement. Each lever press was accompanied by a concurrent illumination of a stimulus light located above the active lever for 2 sec. Each food pellet delivery was followed by a 20 sec time-out period, during which additional active lever presses were recorded but produced no programmed consequences. Presses on the designated inactive lever were recorded but produced no consequences at any time during the experiment. Approximately 24 hr following the initial overnight training session, 3 hr daily heroin self-administration sessions were initiated, whereby presses on the active lever resulted in delivery of heroin (0.03 mg/kg/infusion, delivered in a volume of 0.06 mL over a 2 sec period) on a FR1 schedule of reinforcement. Each drug infusion was followed by a 20 sec time-out period, during which additional active lever presses were recorded but produced no programmed consequences. Each drug infusion was accompanied by concurrent illumination of a stimulus light located above the active lever for 2 sec. Self-administration sessions were conducted 6 consecutive days per week for a total of 12 days. Body weights were assessed every 5–7 days in order to assess potential weight loss as a result of heroin self-administration and to adjust the concentration of heroin in the infusion syringe as needed.

 In Experiment 2, heroin self-administration in GFAP-tk mice was commenced on the day following minipump implantation. Procedures were similar to those of Experiment 1, with the following exceptions: (1) food pellet training was not conducted since mouse self-administration chambers were not equipped with pellet dispensers or receptacles; (2) heroin was delivered in a volume of 0.02 mL over a 1 sec period; (3) self-administration sessions were conducted for a total of 10 days. Group body weights were assessed on the day following minipump implantation and again during each of the 5 extinction days in order to assess potential weight loss as a result of heroin self-administration and/or GCV treatment and to adjust the concentration of heroin in the infusion syringe as needed. BrDU was administered at a dose of 50 mg/kg within 1 minute of the end of each of the 5 extinction sessions.

### 2.6. Extinction Procedures

Extinction sessions were 3 hr in length during which responding on the lever that previously delivered heroin no longer produced any programmed consequences. In addition, the light cue that previously accompanied reinforcer delivery was not available during extinction sessions. Animals were subjected to extinction training to achieve a target level of extinction responding equivalent to 25% of the number of active lever presses that were emitted on the average of the last 2 days of active heroin self-administration. Since the majority of extinction of responding on the active lever in Experiment 1 was observed during the first 5 extinction training sessions, only 5 extinction sessions were conducted in Experiment 2. Immediately following each of the 5 extinction training sessions, mice were injected with BrDU (50 mg/kg i.p.) to provide an indicator of suppression of the formation of immature neurons. 

 In Experiment 1, animals undergoing forced abstinence instead of extinction training were transported to a neutral room at approximately the same time of day as when extinction sessions were conducted, handled briefly, and left undisturbed in the home cage for 3 hr (equivalent to the length of extinction sessions). Rats were then returned to the colony room. This process was repeated daily for a total of 10 days to parallel the number of days that extinction training was conducted.

### 2.7. Assessment of DCX Immunoreactivity

**Figure 2:**
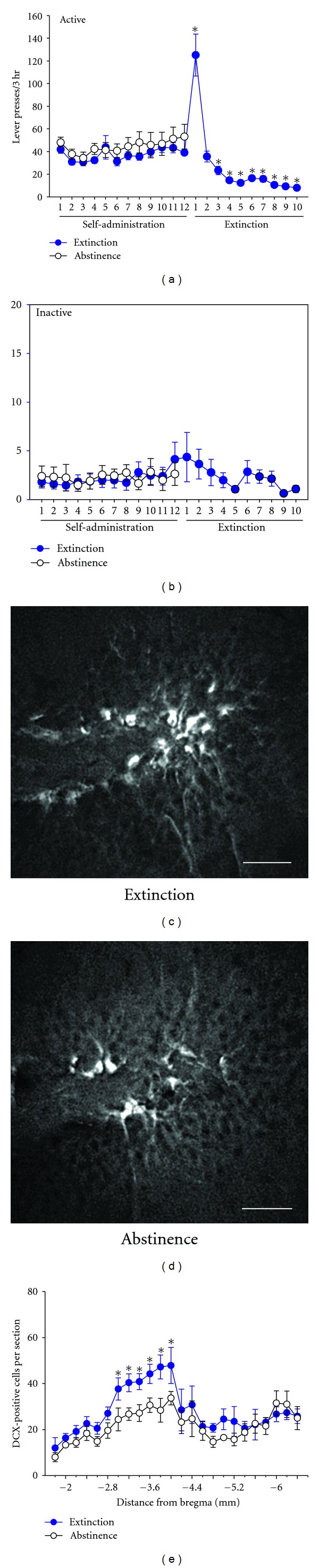
Extinction training increases DCX immunoreactivity in the DG following heroin self-administration (Experiment 1). (a) Active and (b) inactive lever presses during heroin self-administration and extinction. Animals undergoing extinction (*n* = 15) were subjected to 10 days of extinction training following the last day of heroin self-administration, while animals undergoing forced abstinence (*n* = 13) remained in the home cage. *P < 0.05 versus the average of the last two days of heroin self-administration. (c) and (d) Representative DCX immunostaining at 400x magnification in the DG (~3.8 mm posterior to bregma) from animals subjected to extinction (c) or abstinence (d). Scale bar = 35 *μ*m. (e) Quantification of DCX immunoreactivity along the DG neuraxis in a subset of animals subjected to extinction or abstinence (*n* = 8 per group). *P < 0.05 versus the abstinence group at the same coronal plane relative to bregma.

In Experiment 1, rats were anesthetized with sodium pentobarbital (150 mg/kg i.p.) 7 days following the final extinction training session (or on the same day for animals undergoing forced abstinence—see Figure 1) and perfused transcardially with 100 mL of phosphate-buffered saline (PBS, pH = 7.4) containing 0.1% w/v heparin followed by 200 mL of 4% w/v paraformaldehyde (pH = 7.4) in 0.1 M PBS. Brains were removed, postfixed in the fixative solution for 24 hr at 4°C, cryoprotected in 30% w/v sucrose in PBS for 48 hr at 4°C, cut into 30 *μ*m coronal sections along the hippocampal neuraxis), and processed for free-floating DCX immunohistochemistry. Sections were preblocked for 1 hr in PBS containing 0.1% Tween 20, 1 M glycine, and 5% w/v donkey serum, followed by overnight incubation with rabbit anti-DCX primary antisera (1 : 500; Abcam; Cambridge, MA, USA) at 4°C. On the next day, sections were washed, incubated with DyLight 594-conjugated donkey anti-rabbit secondary antisera (1 : 500; Jackson ImmunoResearch; West Grove, PA, USA), washed, mounted onto microscope slides, and coverslipped with Prolong antifade mounting medium (Invitrogen; Carlsbad, CA, USA). Slides were stored in darkness until quantification, which was performed under epifluorescence microscopy at 400x magnification (Leica Microsystems; Bannockburn, IL, USA) by an investigator blind to experimental condition. Manual rotation of the fine focus in the *z*-place was performed to verify the presence of DCX-positive cell bodies. The number of DCX immunoreactive neurons in the subgranular layer of the DG was quantified in one hemisphere from −1.8 to −6.4 mm posterior to bregma to the nearest 0.2 mm according to [32]. Total DCX counts throughout the hippocampal neuraxis were calculated by summation of all counts at each coronal level of section for each individual animal. Images of DCX immunoreactivity in the DG that are presented in Figure 2 were obtained on a Zeiss LSM 410 confocal microscope.

 Immunohistochemical staining of tissue from GFAP-tk mice for DCX immunoreactivity yielded inadequate and unsatisfactory staining patterns, as evidenced by a lack of staining of dendritic processes in the granule cell layer. Use of primary antisera from a different vendor also produced unsatisfactory results. We therefore attempted to verify reductions in neuron production in the DG of these mice induced by GCV using previously published procedures for BrDU immunohistochemistry [33] throughout the mouse hippocampal neuraxis. 

### 2.8. Statistical Analyses

Active and inactive lever press data were analyzed by a one-way ANOVA followed by the Holm-Sidak post hoc tests. DCX immunoreactivity quantified along the hippocampal neuraxis was analyzed by a two-way ANOVA (with the treatment group as the intersubject variable and plane of section as the intrasubject variable) followed by the Holm-Sidak post hoc tests. Total DCX counts in animals undergoing extinction versus abstinence, as well as body weights of mice treated with either saline or GCV, were analyzed by one-way ANOVA. The level of statistical significance was set to *P* < 0.05 for all tests. 

## 3. Results

 The timelines for behavioral procedures in Experiments 1 and 2 are presented in Figure 1. Heroin self-administration data and the effects of extinction versus abstinence on DCX immunoreactivity are depicted in Figure 2. No differences in the number of active or inactive lever presses during active heroin self-administration were observed between animals that subsequently underwent extinction or abstinence (*P* > 0.05). In addition, the number of heroin infusions obtained in each session did not differ between groups (*P* > 0.05, data not shown), indicating that heroin intake was equivalent prior to extinction. Animals subjected to extinction training displayed significant changes in active lever pressing behavior across extinction sessions (*F*(10,133) = 30.2, *P* < 0.001; Figure 2(a)). Presses on the active lever were significantly increased on the first day of extinction as compared to the average of the last 2 days of active heroin self-administration, characteristic of an expected “extinction burst” following removal of heroin as the reinforcer. After the 2nd day of extinction, lever pressing declined and was significantly lower during the 3rd through 10th extinction sessions as compared to the average of the last two days of self-administration. The number of inactive lever presses did not differ across experimental groups and also did not change across extinction sessions (*P*'s > 0.05; Figure 2(b)). The lack of reduction of inactive lever presses produced by extinction training was likely due to a floor effect since the mean number of inactive lever presses per session during the final two days of heroin self-administration was less than 5 for the two groups. 

Analysis of DCX immunoreactivity along the hippocampal neuraxis in animals that underwent either extinction training or abstinence following heroin self-administration revealed that extinction-trained animals showed significantly higher levels of DCX immunoreactivity (*F*(1,208) = 21.95, *P* < 0.001; Figures 2(c)–2(e)) in dorsal regions of the DG ranging from 3.0 to 4.0 mm posterior to bregma. No group differences were detected in ventral/posterior regions of the DG. In addition, total DCX counts throughout all sections analyzed were higher (*F*(1,13) = 8.19, *P* < 0.05) in animals that underwent extinction (502.4 ± 45.6, mean ± SEM) than those that underwent abstinence (357.2 ± 25.8, mean ± SEM).

**Figure 3:**
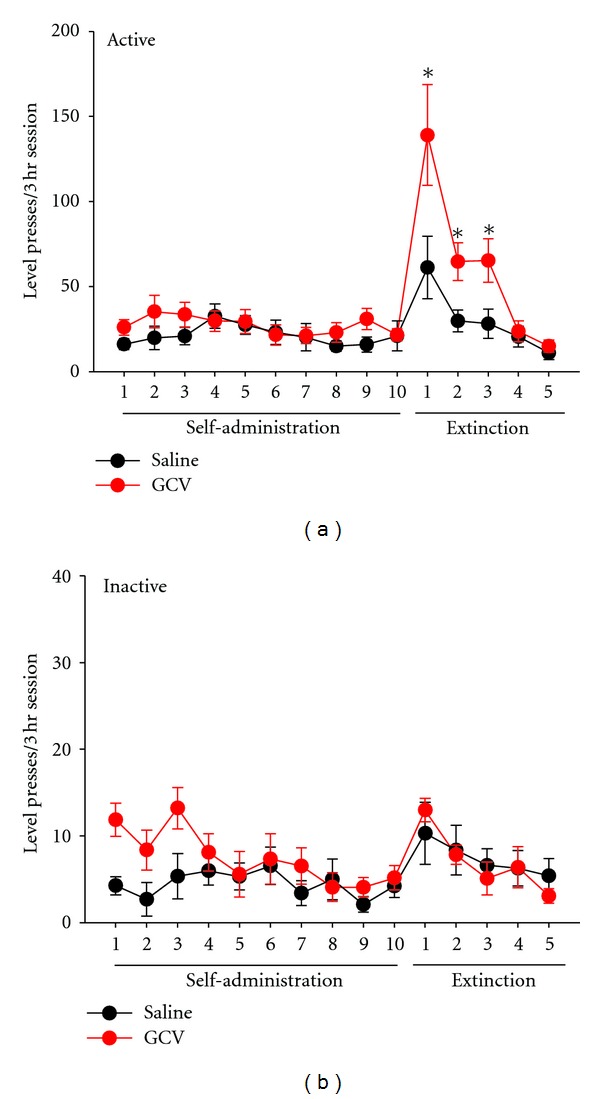
Heroin self-administration and extinction in GFAP-tk mice implanted with minipumps containing either saline or GCV. (a) Active lever presses during 10 days of heroin self-administration followed by 5 days of extinction. Mice treated with GCV (*n* = 11) displayed increased extinction responding during the first 3 extinction days (E1–E3) as compared to mice that were treated with saline (*n* = 9). *P < 0.05 versus saline-treated mice on the same day of extinction. (b) Inactive lever presses during 10 days of heroin self-administration followed by 5 days of extinction.

 The results of Experiment 2 are depicted in Figure 3. No differences in lever pressing during the 10 days of active heroin self-administration were noted in GFAP-tk mice receiving saline or GCV (*P* > 0.05; Figure 3(a)) or in the number of heroin infusions obtained (*P* > 0.05, data not shown) indicating equal levels of heroin intake between the two groups. Levels of heroin intake in these GFAP-tk mice were similar to those reported previously in Balb/c mice [34]. On the first 3 days of extinction, GCV-treated mice displayed an increased number of presses on the lever that previously delivered heroin (*F*(1,226) = 5.8, *P* < 0.05; Figure 3(a)), suggesting an impairment in extinction learning. No differences between treatment groups were noted for inactive lever presses across any phase of the experiment (*P* > 0.05; Figure 3(b)). Similarly, analysis of body weight data before and after heroin self-administration revealed neither weight loss nor weight gain (*P* > 0.05), suggesting that heroin self-administration and/or GCV treatment did not produce nonspecific effects on food intake in GFAP-tk mice.

As mentioned earlier, use of primary antisera against DCX from two different commercial vendors produced inadequate and unsatisfactory staining patterns. Therefore, to verify the GCV-induced reduction of newly proliferating cells in the DG of GFAP-tk mice, immunohistochemistry for BrDU was performed. The number of BrDU-positive cells per section was significantly lower in mice treated with GCV (5.7 ± 0.6, mean ± SEM) as compared with those treated with saline (14.1 ± 0.8, mean ± SEM) (*F*(1,45) = 74.2, *P* < 0.001). 

## 4. Discussion

 In Experiment 1, we demonstrated that extinction training procedures following heroin self-administration produced increased levels of DCX immunoreactivity in the DG, primarily in more rostral/dorsal regions, as compared to animals that underwent abstinence. Extinction of drug-seeking behavior in rodent models of addiction is typically produced by withholding drug reinforcers and associated cues when animals are performing the same operant task that previously resulted in drug infusions and cue presentation. Although we did not assess the effects of extinction on the production of mature neurons in the DG, our findings are in line with those of Rapanelli and colleagues [35], who found that subjecting rats to an operant conditioning task resulted in increased numbers of mature neurons in the dorsal DG.

 In Experiment 2, we found that impairment of cell proliferation in the DG by administration of GCV to GFAP-tk mice resulted in increases in extinction responding during the first 3 sessions following cessation of heroin availability. Similarly, Noonan and colleagues [36] have previously demonstrated that ablation of both mature and immature neurons in the DG by hippocampal irradiation resulted in increased responding during the first 3 extinction sessions following cocaine self-administration. Unfortunately, however, the present study did not assess the impact of GCV treatment on formation of immature versus mature neurons, and only nonspecific quantification of the number of BrDU-positive cells as an indicator of cell proliferation was performed. Nonetheless the short time frame of GCV administration (10 days followed by commencement of extinction training on the following day) and the known amount of time required for the formation of mature neurons in the adult mouse DG (>4 weeks, see [37, 38]), our findings suggest that immature neurons in the DG may play a role in the process of extinction learning. Additional studies using transgenic lines of mice that more precisely allow the examination of the function on DG immature neurons, such as nestin-tk mice [24], are clearly warranted to confirm this.

 We noted that extinction training following heroin self-administration increased DCX immunoreactivity primarily in dorsal regions of the DG, while no such effects were observed in the ventral DG. Numerous lines of evidence suggest that dorsal regions of the hippocampus mediate drug-seeking behavior [39–41], although the precise role of adult-born neurons specifically in the DG, both immature and mature, has not yet been fully defined [17–19]. In contrast to our findings, other investigators have reported that cocaine self-administration followed by either continued drug intake or withdrawal results in preferential increases in DCX immunoreactivity in posterior/ventral regions of the DG [42]. The reasons underlying our observations of extinction-induced increases in DCX immunoreactivity in dorsal DG regions are currently unclear but may be related to the functional dichotomy of dorsal versus ventral areas of the hippocampus, with more dorsal regions mediating spatial information processing and more posterior/ventral regions mediating limbic functions such as emotionality [43]. Future studies employing methods to manipulate immature DG neurons with precise anatomical specificity are needed to resolve this issue.

 There are several limitations of the present study. First, the number of DCX-positive neurons per coronal section in Experiment 1 is lower than those that have been reported by others [36, 42, 44]. There are numerous factors that could have contributed to these differences, such as opiate-induced suppression of NPC proliferation or survival [12, 45–47], different antigens to which DCX primary antisera were raised, and different methods of quantification (stereology versus manual counting). Second, due to technical problems with utilizing DCX antisera in mouse tissue, we were unable to quantify the degree to which GCV suppressed the formation of DCX-immunoreactive immature neurons in the DG, and only a nonspecific assessment of the number of proliferating cells by BrDU immunohistochemistry was performed. Clearly, future studies should be conducted to assess changes in a range of markers of NPC proliferation, differentiation, maturation, and cell death in the DG that occur as a result of extinction training. Third, both mice and rats self-administered heroin under limited access conditions (3 hr/day) and were not likely opiate dependent, which is typically achieved under conditions of extended daily access (6+ hr/day). The effects of extinction training on DCX immunoreactivity in the DG in heroin-dependent animals is an avenue worthy of further exploration.

 In summary, we present evidence that extinction training following heroin self-administration increases the number of DCX-positive immature neurons selectively in the dorsal regions of the DG. We also present evidence that inhibition of cell proliferation in the DG increases responding during extinction, which is indicative of impaired extinction learning. These findings lend further support to the notion that immature neurons in the DG may play a functional role in certain learning and memory processes, including the extinction of drug-seeking behavior.
